# Prevalence and Antimicrobial Resistances of *Salmonella* spp. Isolated from Wild Boars in Liguria Region, Italy

**DOI:** 10.3390/pathogens10050568

**Published:** 2021-05-07

**Authors:** Elisabetta Razzuoli, Valeria Listorti, Isabella Martini, Laura Migone, Lucia Decastelli, Walter Mignone, Enrica Berio, Roberta Battistini, Carlo Ercolini, Laura Serracca, Tiziana Andreoli, Monica Dellepiane, Daniela Adriano, Monica Pitti, Daniela Meloni, Paola Modesto

**Affiliations:** 1 Department of Genoa, Istituto Zooprofilattico Sperimentale del Piemonte, Liguria e Valle d’Aosta, Piazza Borgo Pila 39/24, 16129 Genoa, Italy; isabella.martini@izsto.it (I.M.); laura.migone@izsto.it (L.M.); 2 Department of Turin, Istituto Zooprofilattico Sperimentale del Piemonte, Liguria e Valle d’Aosta, Via Bologna 148, 10154 Turin, Italy; lucia.decastelli@izsto.it (L.D.); daniela.adriano@izsto.it (D.A.); monica.pitti@izsto.it (M.P.); daniela.meloni@izsto.it (D.M.); 3 Department of Imperia, Istituto Zooprofilattico Sperimentale del Piemonte, Liguria e Valle d’Aosta, Via Nizza 4, 18100 Imperia, Italy; walter.mignone@izsto.it (W.M.); enrica.berio@izsto.it (E.B.); 4 Department of La Spezia, Istituto Zooprofilattico Sperimentale del Piemonte, Liguria e Valle d’Aosta, Via degli Stagnoni 96, 19100 La Spezia, Italy; roberta.battistini@izsto.it (R.B.); carlo.ercolini@izsto.it (C.E.); laura.serracca@izsto.it (L.S.); 5 Department of Savona, Istituto Zooprofilattico Sperimentale del Piemonte, Liguria e Valle d’Aosta, Via Martiri 6, 17056 Savona, Italy; tiziana.andreoli@izsto.it (T.A.); monica.dellepiane@izsto.it (M.D.)

**Keywords:** *Salmonella* spp., wild boars, antimicrobic resistance

## Abstract

*Salmonella* spp. is an important zoonotic agent. Wild boars might host this pathogen in the intestinal tract and might represent a risk for *Salmonella* spp. transmission to humans. Wild boars are widely spread in Liguria, due to the environmental characteristics of the region. The aim of the study was the isolation, typing, and investigation of antimicrobial susceptibility of the isolated strains of *Salmonella* spp. During the 2013–2017 hunting seasons, 4335 livers of wild boars were collected and analyzed for the presence of *Salmonella* spp. A total of 260 strains of *Salmonella* spp. were isolated and characterized, with a prevalence of 6%. The isolated strains belonged to all six *Salmonella enterica* subspecies. Most of them were identified as *Salmonella enterica* subs. enterica of which 31 different serotypes were identified. The dominating serotype identified was *S.* Enteritidis. The antimicrobial resistance profiles of the isolated strains were analyzed against sixteen molecules. Of the isolated strains, 94.6% were resistant to at least one of the tested antimicrobials. This study showed the circulation of resistant *Salmonella* spp. strains in the wild boar population living in this area of Italy, underling the potential risk for these animals to disseminate this pathogen and its antimicrobial resistances.

## 1. Introduction

*Salmonella* spp. is one of the most important causes of human zoonosis in Europe [1]. Many serotypes have been isolated from the intestinal tract of a wide range of animals, including wild boars [2], which may act as a reservoir and disseminate pathogenic strains of *Salmonella* spp. [3]. In Europe, the twenty most frequent serotypes of *Salmonella* spp. causing illness in humans as reported by the European Authority for the Food Safety (EFSA), belong to the *S.enterica* subs. enterica, and among them the three most common are *S.* Enteritidis, *S.* Typhimurium, and *S.* Typhimurium monophasic variant 1,4,[5],12:i- [1]. Antimicrobial resistance is one of the most challenging health problems worldwide [4]. Wild boars are an important reservoir of antimicrobial-resistant bacteria and could be used as a sentinel species for surveillance [5]. Antimicrobial-resistant pathogens, including *Salmonella* spp., have been widely isolated from this species [6,7,8]. Wild boars are omnivorous and considering their behavior and habit of rooting through waste in urban areas, sources of exposure to *Salmonella* spp. include the consumption of contaminated carcasses and contact with infected farmed animals [9] or other wild animals. These animals could represent a risk for the transmission of *Salmonella* spp. during evisceration, through consumption of meat and meat products [2], from contamination of vegetables in agricultural areas, and through direct or indirect contact with farmed animals.

Wild boars are widely present in Italy and in Europe with an increasing prevalence [10,11]. In particular, in the area of the survey, the Liguria region, the characteristics of the territory support a wide occurrence of this species as well as in urban and peri-urban areas. In the Liguria region, the presence of wild boars has been recognized as a risk for zoonoses transmission. It has been observed that two important zoonotic agents, *Leptospira* spp. [12] and *Yersinia* spp., circulate in wild boar populations living in this area, and for the second pathogen, the presence of multidrug-resistant strains has been recently observed [13]. The aim of the study was to investigate the presence of *Salmonella* spp. in the liver of wild boars hunted in Liguria region and to analyze the profiles of antibiotic resistance of the isolated strains. This survey was conducted during regional wild animal monitoring plans, from 2013 to 2017. The monitoring plans were carried out in order to investigate the presence of infectious diseases representing a risk for public health in wild boars and other wild animals.

## 2. Results

### 2.1. Salmonella spp. Typing

Of the 4335 samples analyzed, 540 were positive to PCR screening. The positive samples where then cultured and among them a total of 260 *Salmonella* spp. strains were isolated and typed (Table 1), with a prevalence of 6%. Most of the isolated strains (157/260, 60.4%) were typed as *S. enterica* subs. enterica of which a total of 31 serotypes were identified (Table 1). Among them, seven of the twenty most frequent *Salmonella* spp. serotypes responsible for illness in humans in Europe were identified: *S.* Enteritidis (20/260, 7.7%), *S.* Typhimurium (10/260, 3.8%), *S.* Typhimurium monophasic variant 1,4,[5],12:i:- (4/260, 1.5%), *S.* Infantis (3/260, 1.2%), *S.* Newport (8/260, 3.1%), *S.* Napoli (21/260, 8%), and *S.* Coeln (9/260, 3.5%). No clinical signs were reported by hunters and *Salmonella* spp. isolation was not associated with the presence of macroscopic lesions referable to salmonellosis.

### 2.2. Antimicrobial Susceptibility Analysis

The strains were classified as susceptible, intermediate, or resistant. The details of the antimicrobial susceptibility of the isolated strains are reported in Table S1 in the Supplementary Material. The summary concerning the antimicrobial susceptibility of the analyzed strains is reported in Table 2. A total of 94.6% of the analyzed strains (246/260) were resistant to at least one of the tested molecules; 40% (98/260) to two or more; 17.3% (45/260) to three or more; and 9.6% (25/260) of the isolated strains resulted resistant to four of more antimicrobials. In particular, the higher resistances were observed in two strains of *S.* Typhimurium monophasic variant 1,4,[5],12:i:-, three strains of *S.* Brandenburg, and one strain of *S. enterica* subs. salamae that resulted resistant to seven antimicrobials (Table 3). Most of the resistances were observed against sulfadiazine + sulfamerazine + sulfamethazine; in fact 96% of the strains tested resulted resistant to these molecules. Less than the 1% of tested strains resulted resistant to chloramphenicol, colistin, ceftazidime, enrofloxacin, and nalidixic acid. No one strain resulted resistant to ciprofloxacin. The majority of the intermediate susceptibilities were observed against kanamycin (43%), streptomycin (30.2%), and tetracycline (23.4%)

The percentages of strains determined to be antimicrobial resistant, belonging to the most frequent serotypes of *Salmonella* spp. causing human illness in Europe, are reported in Table 4. The strains of *S.* Typhimurium monophasic variant 1,4,[5],12:i:- showed the most noteworthy antimicrobial resistances. In fact, 100% of the analyzed strains showed resistance to: ampicillin, amoxicillin-clavulanic acid, streptomycin, trimethoprim-sulfamethoxazole, and tetracycline; 75% of the analyzed strains showed resistance to cefalotin. The strains of *S.* Coeln showed resistance to nine molecules but in a lower rate. In particular, 100% of the analyzed strains showed resistance to sulfadiazine + sulfamerazine + sulfamethazine; 67% to tetracycline; and 22% to amoxicillin-clavulanic acid, streptomycin, and trimethoprim-sulfamethoxazole. A total of 11% of the analyzed strains resulted resistant to ampicillin, cefalotin, cefotaxime, and ceftazidime. The *S.* Napoli isolated strains showed resistance to four molecules. In particular, 86% of the isolated strains resulted resistant to sulfadiazine + sulfamerazine + sulfamethazine; 19% to ampicillin; 10% to tetracycline; and 5% to streptomycin. The *S.* Typhimurium isolated strains resulted resistant to four molecules. A total of 100% of the analyzed strains resulted resistant to sulfadiazine + sulfamerazine + sulfamethazine; 30% to trimethoprim-sulfamethoxazole; 11% to amoxicillin-clavulanic acid; and 10% to ampicillin. The *S.* Newport isolated strains resulted resistant to three molecules. Among them, 100% of the strains resulted resistant to sulfadiazine + sulfamerazine + sulfamethazine; 63% to streptomycin; and 13% to trimethoprim-sulfamethoxazole. The *S.* Infantis isolated strains resulted resistant to three of the tested molecules. In particular, 100% of the strains resulted resistant to sulfadiazine + sulfamerazine + sulfamethazine and 33% to cefalotin and tetracycline. Also, the *S.* Enteritidis isolated strains resulted resistant to three molecules: 100% was resistant to sulfadiazine + sulfamerazine + sulfamethazine; 25% was resistant to trimethoprim-sulfamethoxazole; and 5% was resistant to tetracycline. Considering these strains in total, the higher intermediate susceptibilities were observed against kanamycin (22%), tetracycline (16.7%), and streptomycin (9.1%).

## 3. Discussion

*Salmonella* spp. is the second cause of human zoonosis in Europe. Many serotypes can be involved in a morbid event causing different clinical signs. The results obtained in our study confirm the possible role of wild boars in the transmission of *Salmonella* spp.

Indeed, the presence of *Salmonella* spp. in the deep part of muscles of wild boars hunted for consumption in Italy was already demonstrated [14] and it was also reported on the carcasses and meat cuts of wild boars in European countries [15]. The presence of *Salmonella* spp. in the liver, that is usually eaten lightly cooked or used for cured meat preparation, suggests the existence of a risk of transmission of *Salmonella* spp. to humans. To our knowledge, no salmonellosis cases following consumption of meat from wild boars have been reported in Italy. This is possibly due to the low prevalence of this pathogen, which was reported in studies conducted in Italy using different matrices (i.e., faeces or intestinal content) [6,7,8]. Another reason could be the low amount of wild animal meat consumed compared with the total amount of meat consumed. In addition, the EFSA reports do not specify the origin of the meat causing human salmonellosis in European countries [1]. However, during an outbreak of *S.* Cholerasuis var. Kusendorf in wild boars in Italy, the same serotype was isolated in a human case of salmonellosis. The phylogenetic and PFGE analyses of the isolated strains suggested a high degree of similarity between the human isolates and the isolates from the wild boar outbreak. This study suggested the potential role of wild boars in spreading *S.* Cholerasuis to humans [16]. However, *Salmonella* spp. can be released from infected wild boars into the environment, causing contamination of surface water, direct or indirect contamination of crops, and could be transmitted to farmed animals. The risk of direct or indirect transmission of *Salmonella* spp. from wild boars to humans is considered possible as previously reported by other authors [2]. Recent studies demonstrated the different ability of *Salmonella* strains to modulate innate immunity; in particular, it was demonstrated that IL-8 expression is very important for *Salmonella* spp. invasion of enterocytes [17,18]. In this respect, Razzuoli and coworkers demonstrated that different strains of *S.* diarizonae isolated in wild boars can have different pathogenicity [18]. Wild boars may act as healthy carriers for *Salmonella* spp. [3], but stressor conditions like the presence of concomitant viral diseases [19] or pollutants such as cadmium [20] could determine a high susceptibility to *Salmonella* spp., and clinical outbreaks could occur [21]. The 6% prevalence of *Salmonella* spp. observed in this study is similar to those reported in previous studies conducted in central Italy [6,8] and northwestern Italy [7], where a prevalence of 4.8%, 7.2%, and 10.8% were reported. *S. enterica* subs. enterica is the most common subspecies isolated in our study. This observation is consistent with other studies conducted in wild boars [3,6,22]. The main location of *Salmonella* spp. in non-adapted hosts is the intestinal tract. The use of liver samples for this study could have led us to underestimate the prevalence of *Salmonella* spp, but the liver was chosen for two reasons. The study was mainly conducted for food hygiene purposes and the liver is an edible part traditionally consumed in this part of Italy and also used for the production of cured meats; therefore, it could represent a risk for the transmission of *Salmonella* spp. to humans. The second reason was a practical trade-off. Sampling was carried out during the evisceration process by the hunters and the other parts of the carcasses were left at the disposal of hunters to then be consumed. In practice, it was better to not use the intestinal content because its collection could have represented a risk of contamination of the carcasses and would not have guaranteed transport in safe hygienic conditions to the laboratory. Other studies have investigated the presence of *Salmonella* spp. in this organ [8].

The other subspecies of *Salmonella* spp. represent 40% of the isolated strains. Among them, *S. enterica* subs. salamae is the most frequently isolated subspecies (20.4%). This subspecies of *Salmonella* has also been isolated in a high rate in an investigation previously conducted in Italy in wild boars [6]. Among the strains most frequently implicated in human illness in Europe [1], our results showed the presence of *S.* Enteritidis, *S.* Typhimurium, *S.* Typhimurium monophasic variant 1,4,[5],12:i:-, *S.* Infantis, *S.* Newport, *S.* Napoli, and *S.* Coeln. These strains were previously reported in wild boars in Italy [3,6,7,8]. The relative high number (21/260, 8%) of isolates belonging to *S.* Napoli serotype is interesting; in fact it is a re-emerging serotype in Italy. It has been hypothesized that the environment can act as reservoir for this serotype from where it can spill over to humans and animals [23]. This serotype has been already isolated form wild boars in Italy [3,24]. Our investigation confirms the circulation of *S.* Napoli in wild boars that can represent a route of dissemination of this serotype in humans and farmed animals, directly or contaminating surface water. Another interesting finding is the isolation of S. Stourbridge, a rare serotype that was involved in some severe outbreaks in Germany in 2016. Human patients presented severe clinical signs and two had a fatal outcome. Unfortunately, the source of infection has not been identified [25]. Following these outbreaks, ECDC asked for collaboration and sharing of data about the identification of *S.* Stourbridge from non-human sources. Indeed, few literature data are available about *S.* Stourbridge isolation, mainly from human patients and cattle [26]. To the extent of our knowledge, this is the first description of *S.* Stourbridge from wild boars in Italy, while it has already been described from a wild boar in Switzerland [27]. The present result suggests that wild boar are probably responsible for the *S.* Stourbridge source of infection. *S.* Cholerasuis is a host-adapted serotype, an agent of swine paratyphoid [28], and in our study, we did not isolate this serotype of *Salmonella.* The hunted wild boars did not show clinical signs and the isolation of *Salmonella* spp. were not associated with the presence of macroscopic lesions. The presence of *S.* Cholerasuis has been mainly reported in domestic pigs [29] and a possible mutual exposure to this pathogen between domestic pigs and wild boars has been hypostatised [30]. In our study, this serotype was not found.

The Liguria region has a very low density of farmed pigs with few animals at each farm site. This led us to hypothesize that contact between farmed pigs and wild boars is improbable, indicating a low probability of spread of this serotype in the wild boar population, despite a limited exchange of *Salmonella* spp. having been observed among domestic pigs and wild boars in Italy [31]. Moreover, the presence of this pathogen, to the extent of our knowledge, was not reported for farmed pigs in this area of Italy, and studies conducted in adjoining regions in wild boars did not detect this serotype too [8]. Wild animals might act as reservoir for antimicrobial resistance pathogens. It has been suggested that wildlife living in urban areas show higher antimicrobial resistances than animals living in remote areas [32], due to the possibility of contact with resistant bacteria and selective agents. Wild boars have contact with humanized environments, thus is a species that could be implicated in the cycle of antimicrobial resistance transmission [5]. In our study we confirm this evidence, showing that almost 94.6% of the isolated *Salmonella* spp. strains are characterized by a resistance to at least one antimicrobial tested. The highest resistances were observed against sulfonamides. In particular, 96% of the tested strains resulted resistant to the combination of sulfadiazine + sulfamerazine + sulfamethazine (triple-sulfa) and 21.9% to the combination of sulfamethoxazole + trimethoprim. Our results confirm the well-known, widespread antimicrobial resistance against these molecules, which, for this reason, have been less used by the clinicians for a long time [33]. A total of 20% of the tested strains resulted resistant to tetracycline; the antimicrobial resistance is well established for this molecule, and the use is limited only in infection with confirmed susceptibility [34]. A total of 10.9% of the isolated strains resulted resistant to streptomycin. Our results are consistent with other studies on the presence of *Salmonella* spp. in wild mammals and in other territories, in which the majority of the resistances reported are against the same classes of antimicrobials [6,7,22,35] and in contrast with the results of a recent study conducted in central Italy in wild boars [8] where no resistances against tetracycline were observed, and a higher rate of antimicrobial resistance to streptomycin was reported. In our study, the antimicrobial susceptibility against molecules considered “Highest Priority Critically Important” antimicrobials for human medicine has been tested, among them, quinolones (nalidixic acid and ciprofloxacin) and cephalosporines of third generation (cefotaxime, ceftazidime). Both of these classes of antimicrobials are known to select for resistant *Salmonella* spp. and *E. coli* strains in animals, and at the same time, are among the few available therapies for serious *Salmonella* spp. and *E. coli* infections [36]. In our study, we also tested the antimicrobial susceptibility against colistin. This molecule belongs to the polymixines, which are known to select for plasmid-mediated polymyxin-resistant *E. coli* in food animals, but at the same time, are one of the few available therapies for serious Enterobactericeae and *Pseudomonas aeruginosa* multi-resistant infections [36]. Fortunately, most of the isolated strains are sensitive to these antimicrobials. Considering the quinolones, only 0.8% of the tested strains resulted resistant to nalidixic acid, and no one strain resulted resistant to ciprofloxacin. The analysis of the antimicrobial susceptibility against cephalosporin of third generation also revealed a low rate of antimicrobial resistance. In particular, 1.2% of the isolated strains resulted resistant to cefotaxime and 0.8% to ceftazidime. Only 0.4% of the isolated strains resulted resistant to colistin.

The observed antimicrobial resistance to these molecules is lower than that reported in other studies conducted in wild boars; however, these studies considered a lower number of *Salmonella* spp. strains [6,8,21]. The majority of the intermediate susceptibility was observed against kanamycin (43%), streptomycin (30.2%), and tetracycline (23.4%). However, considering only the seven serotypes, among the most frequently isolated in human illness in Europe, the percentage of intermediate susceptibility are lower when compared to all tested strains: kanamycin (22%), streptomycin (9.1%), and tetracycline (16.7%). Considering all the tested strains, the highest intermediate susceptibility against the “Highest Priority Critically Important” antimicrobials was observed against ceftazidime (20.9%), followed by nalidixic acid (12.6%), cefotaxime (7.8%), colistin (2.9%), and ciprofloxacin (0.8%). As in the previous case, considering the strains of the seven serotypes, among the most frequently isolated in human illness in Europe, the percentage of intermediate strains was lower: ceftazidime (7.6%), cefotaxime (3%), followed by nalidixic acid (2.3%), and colistin (0.8%); no intermediate susceptibility was observed against ciprofloxacin. Considering these observations in total, we can assert that, despite the presence of multi-drug resistances, the isolated strains present a high sensitivity rate against “Highest Priority Critically Important” antimicrobials in use.

## 4. Materials and Methods

### 4.1. Samples Collection

The survey was conducted during the hunting seasons from 2013–2017 (October to January). During the evisceration of hunted wild boars, 4335 livers were collected and analyzed for the presence of *Salmonella* spp. The area of the sampling was the Liguria region, in the northwest of Italy. The liver of each wild boar was collected by the hunters using dedicated knives and sterile bags provided by the laboratory and within 4 h after collection were transported refrigerated to the laboratory. The hunters were trained by laboratory workers on the procedures for the evisceration.

### 4.2. Salmonella Isolation and Typing

Twenty-five grams of each liver were sampled in the deep part of the organ, enriched (1:10) in buffered peptone water (BPW), homogenized in a Stomacher blender, and incubated for 18 h at 37 ± 1 °C. PCR method was used for screening analysis. The DNA was extracted and amplified using the iQ-Check Salmonella II PCR Detection kit (Bio-rad, Milan, Italy), according to the instructions of the producer. The isolation of Salmonella strains from PCR-positive samples was made according to ISO 6579:2002/COR. 1, 2004 (Microbiology of food and animal feeding stuffs: horizontal method for the detection of *Salmonella* spp.). Briefly, after the pre-enrichment, 100 µL of sample was added to 10 mL of Rappaport Vassiliadis modified broth (RVS), and 1 mL of sample was added to 10 mL of Muller–Kauffmann tetrathionate-novobiocin broth (MKTTn), respectively. RVS broth was incubated for 24 h at 41.5 ± 1 °C and the MKTTn broth for 24 h at 37 ± 1 °C. After the incubation, the samples were distributed onto plates of selective media, brilliant green agar (BGA) and xylose lysine deoxycholate agar (XLD). The plates were incubated for 24 h at 37 ± 1 °C, and then suspected colonies were transferred on nutrient agar and incubated at 37 ± 1 °C for 20–24 h. After this step, the suspected colonies were seeded in triple sugar iron (TSI) to evaluate H_2_S production and glucose, lactose, and sucrose fermentation. The presence of *Salmonella* was confirmed using appropriate biochemical miniaturized tests (API 20E^®^, Biomerieux, Lyon, France) and serological tests. Serotype identification of the isolated strains was carried out according to ISO/TR 6579-3, 2014 (Microbiology of the food chain—Horizontal method for the detection, enumeration and serotyping of *Salmonella*—Part 3: Guidelines for serotyping of *Salmonella* spp.).

### 4.3. Antimicrobial Susceptibility Analysis

The Kirby–Bauer disk diffusion test was performed according to Clinical and Laboratory Standard Institute (CLSI) guidelines [37] using Mueller–Hinton agar plates (Microbiol, Uta, Italy) and following the indications of the Italian National Reference Laboratory. The antimicrobials and concentrations (μg) used were: ampicillin (A, 10), amoxicillin/clavulanic acid 2:1 (AMC, 30), chloramphenicol (C, 30), cefalotin (KF, 30), cefotaxime (CTX, 5), cyprofloxacin (CIP, 5), colistin (CST, 10), ceftazidime (CAZ, 10), enrofloxacin (ENR, 5), gentamicin (G, 10), kanamycin (K, 30), nalidixic acid (NAL, 30), streptomycin (S, 10), triple-sulfa (SSS, 250), trimethoprim-sulfamethoxazole (SXT, 1.25/23.75), and tetracycline (T, 30). The interpretation of results was performed according to the CLSI guideline instructions [38,39,40].

## 5. Conclusions

Continuous monitoring of the prevalence and antimicrobial resistance profiles of *Salmonella* spp. on wild boars is useful for the epidemiological surveillance on territories, by means of animal sentinels. This kind of monitoring is also important for risk analysis on the consumption of meat and meat products of this species, which is traditionally consumed in our region as well as in many parts of Italy. In conclusion, the study underlines the possible role of wild boars in the diffusion of *Salmonella* spp. Moreover, due to the antimicrobial resistance found in our study, a risk in the spread of antimicrobial resistant strains can be supposed. Considering wild boars as sentinel, we can assert the low presence of antimicrobial resistance against “Highest Priority Critically Important” antimicrobials in the territory of the survey area.

## Figures and Tables

**Table 1:** Subspecies of *S. enterica* and serotypes of *Salmonella enterica* subs. *enterica* isolated.

Isolated Serotypes or Subspecies	Number/260	Percentage/Tot.
*S. enterica* subs. enterica (not serotyped)	5	1.9%
*S.* Enteritidis	20	7.7%
*S.* Typhimurium	10	3.8%
*S.* Typhiumurium monophasic variant 1,4,[5],12:i:-	4	1.5%
*S.* Infantis	3	1.2%
*S.* Newport	8	3.1%
*S.* Napoli	21	8%
*S.* Coeln	9	3.5%
*S.* Brandenburg	3	1.2%
*S.* Veneziana	10	3.8%
*S.* Thompson	8	3.1%
*S.* Canada	8	3.1%
*S.* Oxford	7	2.7%
*S.* Muenster	6	2.3%
*S.* Kottbus	5	1.9%
*S.* Galil	4	1.5%
*S.* Kimuenza	6	2.3%
*S.* Banjul	4	1.5%
*S.* Stourbridge	3	1.2%
*S.* Juba	2	0.8%
*S.* Arechavaleta	2	0.8%
*S.* Atakpame	1	0.4%
*S.* Stoneferry	1	0.4%
*S.* Umbilo	1	0.4%
*S.* Goldocoast	1	0.4%
*S.* Grampiam	1	0.4%
*S.* Ablogame	1	0.4%
*S.* Massakory	1	0.4%
*S.* Bispebjerg	1	0.4%
*S.* Bahrenfeld	1	0.4%
*S. enterica* subs. salamae	53	20.4%
*S. enterica* subs. arizonae	13	5%
*S. enterica* subs. diarizonae	29	11.2%
*S. enterica* subs. houtenae	7	2.7%
*S. enterica* subs. indica	1	0.4%

**Table 2:** Percentage of isolated strain which resulted susceptible, intermediate, or resistant to the tested antimicrobials.

Antibiotic	Susceptible	Intermediate	Resistant
Ampicillin	206/260 (79.2%)	29/260 (11.2%)	25/260 (9.6%)
Amoxicillin + Clavulanic Acid	171/217 (78.8%)	33/217 (15.2%)	13/217 (6%)
Chloramphenicol	247/251 (98.4%)	3/251 (1.2%)	1/251 (0.4%)
Cefalotin	197/260 (75.8%)	47/260 (18%)	16/260 (6.2%)
Cefotaxime	235/258 (91%)	20/258 (7.8%)	3/258 (1.2%)
Ciprofloxacin	258/260 (99.2%)	2/260 (0.8%)	0/260 (0%)
Colistin	237/245 (96.7%)	7/245 (2.9%)	1/245 (0.4%)
Ceftazidime	195/249 (78.3%)	52/249 (20.9%)	2/249 (0.8%)
Enrofloxacin	190/207 (91.8%)	16/207 (7.7%)	1/207 (0.5%)
Gentamicin	223/257 (86.8%)	27/257 (10.5%)	7/257 (2.7%)
Kanamycin	118/230 (51.3%)	99/230 (43%)	13/230 (5.7%)
Nalidixic acid	219/253 (86.6%)	32/253 (12.6%)	2/253 (0.8%)
Streptomycin	106/258 (41%)	78/258 (30.2%)	28/258 (10.8%)
Sulfadiazine + Sulfamerazine + Sulfamethazine	6/248 (2.4%)	4/248 (1.6%)	238/248 (96%)
Sulfamethoxazole + Trimethoprim	153/260 (58.8%)	50/260 (19.2%)	57/260 (21.9%)
Tetracycline	147/260 (56.5%)	61/260 (23.4%)	52/260 (20%)

**Table 3:** Antimicrobial resistance profile of multidrug-resistant strains (to 4 or more antimicrobials). In bold: antimicrobials considered “Highest Priority Critically Important”.

Year	Strain	Antimicrobial Resistances
2014	*S.* Typhimurium monophasic variant 1,4,[5],12:i:-	A-AMC-KF-S-SSS-SXT-T
2014	*S.* Typhimurium monophasic variant 1,4,[5],12:i:-	A-AMC-KF-S-SSS-SXT-T
2014	*S.* Typhimurium monophasic variant 1,4,[5],12:i:-	A-AMC-KF-S-SXT-T
2014	*S.* Typhimurium monophasic variant 1,4,[5],12:i:-	A-AMC-S-SSS-SXT-T
2013	*S.* Napoli	A-S-SSS-T
2013	*S.* Coeln	KF-**CST-CAZ**-S-SSS-T
2015	*S.* Coeln	S-SSS-SXT-T
2013	*S.* Brandenburg	A-AMC-KF-**CTX**-K-SSS-T
2013	*S.* Brandenburg	A-AMC-KF-**CTX**-K-SSS-T
2013	*S.* Brandenburg	A-AMC-KF-**CTX**-K-SSS-T
2013	*S.* Stourbridge	S-SSS-SXT-T
2013	*S.* Stourbridge	S-SSS-SXT-T
2013	*S.* Muenster	A-AMC-KF-SSS
2013	*S.* Atakpame	A-AMC-KF-SSS-SXT-T
2016	*S.* Veneziana	G-K-S-SSS-SXT-T
2016	*S.* Veneziana	G-K-SSS-SXT-T
2016	*S.* Canada	A-SSS-SXT-T
2016	*S.* Bahrenfeld	G-K-SSS-SXT-T
2013	*S. enterica* subs. salamae	A-AMC-C-KF-SSS-SXT-T
2014	*S. enterica* subs. salamae	A-KF-SSS-T
2016	*S. enterica* subs. salamae	A-KF-S-SSS-SXT-T
2016	*S. enterica* subs. salamae	G-K-S-SSS
2016	*S. enterica* subs. salamae	ENR-G-K-SSS-SXT
2016	*S. enterica* subs. salamae	G-K-SSS-SXT-T
2014	*S. enterica* subs. diarizonae	S-SSS-SXT-T

A: ampicillin, AMC: amoxicillin/clavulanic acid 2:1, C: chloramphenicol, KF: cefalotin, CTX: cefotaxime, CST: colistin, CAZ: ceftazidime, ENR: enrofloxacin, G: gentamicin, K: kanamycin, S: streptomycin, SSS: Sulfadiazine + Sulfamerazine + Sulfamethazine, SXT: trimethoprim-sulfamethoxazole, T: tetracycline.

**Table 4:** Percentages of tested strains which showed antimicrobial resistances belonging to the most frequent serotypes of *Salmonella* spp. causing human illness in Europe. In bold: antimicrobials considered “Highest Priority Critically Important”.

**Serotype**	**Antimicrobials**															
	A	AMC	C	KF	**CTX**	**CIP**	**CST**	**CAZ**	ENR	G	K	**NAL**	S	SSS	SXT	T
*S.* Enteritidis	- *	-	-	-	-	-	-	-	-	-	-	-	-	20/20 (100%)	5/20 (25%)	1/20 (5%)
*S.* Typhimurium	1/10 (10%)	1/9 (11%)	-	-	-	-	-	-	-	-	-	-	-	6/6 (100%)	3/10 (30%)	-
*S.* Typhimurium monoph. variant	4/4 (100%)	4/4 (100%)	-	3/4 (75%)	-	-	-	-	-	-	-	-	4/4 (100%)	3/3 (100%)	4/4 (100%)	4/4 (100%)
*S.* Infantis	-	-	-	1/3 (33%)	-	-	-	-	-	-	-	-	-	3/3 (100%)	-	1/3 (33%)
*S.* Newport	-	-	-		-	-	-	-	-	-	-	-	5/8 (63%)	8/8 (100%)	1/8 (13%)	-
*S.* Napoli	4/21 (19%)	-	-	-	-	-	-	-	-	-	-	-	1/21 (5%)	18/21 (86%)	-	2/21 (10%)
*S.* Coeln	1/9 (11%)	2/9 (22%)	-	1/9 (11%)	-	-	**1/9** **(11%)**	**1/9** **(11%)**	-	-	-	-	2/9 (22%)	9/9 (100%)	2/9 (22%)	6/9 (67%)

A: ampicillin, AMC: amoxicillin/clavulanic acid 2:1, C: chloramphenicol, KF: cefalotin, CTX: cefotaxime, CIP: ciprofloxacin, CST: colistin, CAZ: ceftazidime, ENR: enrofloxacin, G: gentamicin, K: kanamycin, NAL: nalidixic acid, S: streptomycin, SSS: Sulfadiazine + Sulfamerazine + Sulfamethazine, SXT: trimethoprim-sulfamethoxazole, T: tetracycline. - *: Resistance not observed.

## Supplementary Materials

The following are available online at https://www.mdpi.com/article/10.3390/pathogens10050568/s1, Table S1: Progressive number, lab. code, year of isolation, subtype or serotype, and antimicrobial susceptibility of isolated samples.

## References

[1] European Food Safety Authority (EFSA), European Centre for Disease Prevention and Control (ECDC) (2021). The European Union One Health 2019 Zoonoses Report. EFSA J..

[2] Vieira-Pinto M., Morais L., Caleja C., Themudo P., Torres C., Igrejas G., Poeta P., Martins C. (2011). *Salmonella* sp. in game (*Sus scrofa* and *Oryctolagus cuniculus*). Foodborne Pathog. Dis..

[3] Chiari M., Zanoni M., Tagliabue S., Lavazza A., Alborali L.G. (2013). *Salmonella* serotypes in wild boars (*Sus scrofa*) hunted in northern Italy. Acta Vet. Scand..

[4] WHO (2014). Antimicrobial Resistance: Global Report on Surveillance.

[5] Torres R.T., Fernandes J., Carvalho J., Cunha M.V., Caetano T., Mendo S., Serrano E., Fonseca C. (2020). Wild boar as a reservoir of antimicrobial resistance. Sci. Total Environ..

[6] Zottola T., Montagnaro S., Magnapera C., Sasso S., De Martino L., Bragagnolo A., D’Amici L., Condoleo R., Pisanelli G., Iovane G. (2013). Prevalence and antimicrobial susceptibility of Salmonella in European wild boar (*Sus scrofa*); Latium Region—Italy. Comp. Immunol. Microbiol. Infect. Dis..

[7] Botti V., Navillod F.V., Domenis L., Orusa R., Pepe E., Robetto S., Guidetti C. (2013). *Salmonella* spp. and antibiotic-resistant strains in wild mammals and birds in north-western Italy from 2002 to 2010. Vet. Ital..

[8] Cilia G., Turchi B., Fratini F., Bilei S., Bossù T., De Marchis M.L., Cerri D., Pacini M.I., Bertelloni F. (2021). Prevalence, Virulence and Antimicrobial Susceptibility of *Salmonella* spp.; *Yersinia enterocolitica* and *Listeria monocytogenes* in European Wild Boar (*Sus scrofa*) Hunted in Tuscany (Central Italy). Pathogens.

[9] Navarro-Gonzalez N., Mentaberre G., Porrero C.M., Serrano E., Mateos A., López-Martín J.M., Lavín S., Domínguez L. (2012). Effect of cattle on *Salmonella* carriage, diversity and antimicrobial resistance in free-ranging wild boar (*Sus scrofa*) in northeastern Spain. PLoS ONE.

[10] Massei G., Kindberg J., Licoppe A., Gačić D., Šprem N., Kamler J., Baubet E., Hohmann U., Monaco A., Ozoliņš J. (2015). Wild boar populations up, numbers of hunters down? A review of trends and implications for Europe. Pest. Manag. Sci..

[11] Pittiglio C., Khomenko S., Beltran-Alcrudo D. (2018). Wild boar mapping using population-density statistics: From polygons to high resolution raster maps. PLoS ONE.

[12] Cilia G., Bertelloni F., Mignone W., Spina S., Berio E., Razzuoli E., Vencia W., Franco V., Cecchi F., Bogi S. (2020). Molecular detection of *Leptospira* spp. in wild boar (*Sus scrofa*) hunted in Liguria region (Italy). Comp. Immunol. Microbiol. Infect. Dis..

[13] Modesto P., De Ciucis C.G., Vencia W., Pugliano M.C., Mignone W., Berio E., Masotti C., Ercolini C., Serracca L., Andreoli T. (2021). 2021 Evidence of antimicrobial resistance and presence of pathogenicity genes in *Yersinia enterocolitica* isolate from wild boars. Pathogens.

[14] Decastelli L., Giaccone V., Mignone W. (1995). Bacteriological examination of meat of wild boars shot down in Piedmont and Liguria, Italy. IBEX J. Mt. Ecol..

[15] Paulsen P., Smulders F.J.M., Hilbert F. (2012). *Salmonella* in meat from hunted game: A Central European perspective. Food Res. Int..

[16] Longo A., Losasso C., Vitulano F., Mastrorilli E., Turchetto S., Petrin S., Mantovani C., Dalla Pozza M.C., Ramon E., Conedera G. (2019). Insight into an outbreak of *Salmonella* Choleraesuis var. Kunzendorf in wild boars. Vet. Microbiol..

[17] Chirullo B., Pesciaroli M., Drumo R., Ruggeri J., Razzuoli E., Pistoia C., Petrucci P., Martinelli N., Cucco L., Moscati L. (2015). *Salmonella Typhimurium* exploits inflammation to its own advantage in piglets. Front. Microbiol..

[18] Razzuoli E., Amadori M., Lazzara F., Bilato D., Ferraris M., Vito G., Ferrari A. (2017). *Salmonella* serovar-specific interaction with jejunal epithelial cells. Vet. Microbiol..

[19] Wills R.W., Gray J.T., Fedorka-Cray P.J., Yoon K.J., Ladely S., Zimmerman J.J. (2000). Synergism between porcine reproductive and respiratory syndrome virus (PRRSV) and *Salmonella cholerasuis* in swine. Vet. Microbiol..

[20] Razzuoli E., Mignone G., Lazzara F., Vencia W., Ferraris M., Masiello L., Vivaldi B., Ferrari A., Bozzetta E., Amadori M. (2018). Impact of cadmium exposure on swine enterocytes. Toxicol. Lett..

[21] Gil Molino M., Risco Pérez D., Gonçalves Blanco P., Fernandez Llario P., Quesada Molina A., García Sánchez A., Cuesta Gerveno J.M., Gómez Gordo L., Martín Cano F.E., Pérez Martínez R. (2019). Outbreaks of antimicrobial resistant *Salmonella Choleraesuis* in wild boars piglets from central-western Spain. Transbound. Emerg. Dis..

[22] Gil Molino M., García Sánchez A., Risco Pérez D., Gonçalves Blanco P., Quesada Molina A., Rey Pérez J., Martín Cano F.E., Cerrato Horrillo R., Hermoso-de-Mendoza Salcedo J., Fernández Llario P. (2019). Prevalence of *Salmonella* spp. in tonsils, mandibular lymph nodes and faeces of wild boar from Spain and genetic relationship between isolates. Transbound. Emerg. Dis..

[23] Graziani C., Busani L., Dionisi A.M., Caprioli A., Ivarsson S., Hedenström I., Luzzi I. (2011). Virulotyping of *Salmonella enterica* serovar Napoli strains isolated in Italy from human and nonhuman sources. Foodborne Pathog. Dis..

[24] Sabbatucci M., Dionisi A.M., Pezzotti P., Lucarelli C., Barco L., Mancin M., Luzzi I. (2018). Molecular and Epidemiologic Analysis of Re emergent *Salmonella enterica* Serovar Napoli, Italy, 2011–2015. Emerg. Infect. Dis..

[25] ECDC European Centre for Disease Prevention and Control (2016). Increase in Salmonella Stourbridge Infections in Germany during 2016.

[26] ECDC European Centre for Disease Prevention and Control (2017). Increase in Number of Salmonella Stourbridge Infections in Germany during 2016.

[27] Wacheck S., Fredriksson-Ahomaa M., König M., Stolle A., Stephan R. (2010). Wild boars as an important reservoir for foodborne pathogens. Foodborne Pathog. Dis..

[28] Wilcock B.P., Schwartz K., Leman A.D., Straw B.E., Mengeling W.E., D’Allaire S., Taylor D.J. (1992). Salmonellosis. Diseases in Swine.

[29] Gray J.T., Fedorka-Cray P.J., Stabel T.S., Kramer T.T. (1996). Natural transmission of *Salmonella choleraesuis* in swine. Appl. Environ. Microbiol..

[30] Methner U., Heller M., Bocklisch H. (2009). *Salmonella enterica* subspecies *enterica* serovar Choleraesuis in a wild boar population in Germany. Eur. J. Wildl. Res..

[31] Bonardi S., Bolzoni L., Zanoni R.G., Morganti M., Corradi M., Gilioli S., Pongolini S. (2019). Limited Exchange of *Salmonella* among Domestic Pigs and Wild Boars in Italy. Ecohealth.

[32] Radhouani H., Silva N., Poeta P., Torres C., Correia S., Igrejas G. (2014). Potential impact of antimicrobial resistance in wildlife, environment and human health. Front. Microbiol..

[33] Sköld O. (2000). Sulfonamide resistance: Mechanisms and trends. Drug Resist. Updates.

[34] Grossman T.H. (2016). Tetracycline Antibiotics and Resistance. Cold Spring Harb. Perspect. Med..

[35] Caleja C., de Toro M., Gonçalves A., Themudo P., Vieira-Pinto M., Monteiro D., Rodrigues J., Sáenz Y., Carvalho C., Igrejas G. (2011). Antimicrobial resistance and class I integrons in *Salmonella enterica* isolates from wild boars and Bísaro pigs. Int. Microbiol..

[36] WHO https://www.who.int/foodsafety/publications/antimicrobials-sixth/en/.

[37] CLSI Document M02-A11 (2012). Performance Standards for Antimicrobial Disk Susceptibility Tests.

[38] CLSI Document M100-S17 (2007). Performance Standards for Antimicrobial Susceptibility Testing: Seventeenth Informational Supplement.

[39] CLSI Document M100-S22 (2012). Performance Standards for Antimicrobial Susceptibility Testing: Twenty-Second Informational Supplement.

[40] CLSI Document M100-S23 (2014). Performance Standards for Antimicrobial Susceptibility Testing: Twenty-Third Informational Supplement.

