# Supplementary material for: Prevalence and Antimicrobial Resistances of Salmonella spp. Isolated from Wild Boars in Liguria Region, Italy
Source: Pathogens. 2021 May 7;10(5):568. doi: 10.3390/pathogens10050568 (PMC8151295; doi:10.3390/pathogens10050568)
Supplement: Supplementary file 1 [file pathogens-10-00568-s001.zip › pathogens-1186864-supplementary.pdf]

Table S1. Progressive number, lab. code, year of isolation, subtype or serotype and antimicrobial susceptibility of isolated samples

n.	Lab cod.	YEAR	SUBTYPE or SEROTYPE	A	AMC	C	KF	CTX	CIP	CST	CAZ	ENR	G	K	NAL	S	SSS	SXT	T
1	108932	2013	S. enterica subs. enterica	I	S	S	S	S	S	S	S	S	S	I	S	S	I	S	S
2	108919	2013	S. enterica subs. enterica	S	S	S	S	S	S	S	S	S	S	S	S	S	S	S	S
3	108918	2013	S. enterica subs. enterica	I	S	S	S	S	S	S	S	S	S	S	S	S	R	S	S
4	89427 conf. 3/1	2014	S. enterica subs. enterica	S	S	S	I	S	S	S	I	*n.t.	S	n.t.	I	S	R	I	I
5	89427 conf.3/2	2014	S. enterica subs. enterica	S	S	S	I	S	S	S	I	n.t.	S	n.t.	I	S	R	I	I
6	59218	2013	S. Enteritidis	S	I	S	S	S	S	S	I	I	S	S	S	S	R	S	I
7	76452	2013	S. Enteritidis	S	S	S	S	S	S	S	I	S	S	I	S	I	R	S	I
8	114555 conf.2/5	2014	S. Enteritidis	S	I	S	S	S	S	S	S	S	S	S	S	S	R	S	S
9	114555 CONF. 3/3	2014	S. Enteritidis	S	n.t.	S	S	S	S	S	S	S	S	S	S	S	R	S	S
10	114555 CONF. 3/5	2014	S. Enteritidis	S	n.t.	S	S	S	S	S	S	S	S	S	S	S	R	S	S
11	114555 CONF. 3/6	2014	S. Enteritidis	S	n.t.	S	S	S	S	S	S	S	S	S	S	S	R	S	S
12	114555 CONF. 3/7	2014	S. Enteritidis	S	n.t.	S	S	S	S	S	S	S	S	S	S	S	R	S	S
13	114555 CONF. 3/4	2014	S. Enteritidis	S	S	S	S	S	S	S	S	S	S	S	S	S	R	S	S
14	114555 CONF. 3/8	2014	S. Enteritidis	S	I	S	S	S	S	S	S	S	S	I	S	S	R	S	S
15	114555 CONF. 3/9	2014	S. Enteritidis	S	n.t.	S	S	S	S	S	S	S	S	I	S	S	R	S	S
16	114555 CONF. 4/4	2014	S. Enteritidis	S	n.t.	S	S	S	S	S	S	S	S	S	S	S	R	S	S
17	114555 CONF. 4/5	2014	S. Enteritidis	S	n.t.	S	S	S	S	S	S	S	I	S	S	I	R	I	S
18	114555 CONF.5/5	2014	S. Enteritidis	S	n.t.	S	S	S	S	S	S	S	S	S	S	S	R	S	S
19	114555 CONF.5/6	2014	S. Enteritidis	S	n.t.	S	S	S	S	S	S	S	S	S	S	S	R	I	S
20	7378 conf. 2/2	2016	S. Enteritidis	S	S	S	S	S	S	S	S	S	S	I	S	S	R	I	I
21	7378 conf. 3/3	2016	S. Enteritidis	S	S	S	S	S	S	S	S	S	I	I	S	S	R	R	R
22	7378 conf.4/1	2016	S. Enteritidis	S	S	S	S	S	S	S	S	S	S	I	S	S	R	R	I
23	7378 conf.4/3	2016	S. Enteritidis	S	S	S	S	S	S	S	S	S	S	I	S	S	R	R	S
24	7378 conf.4/4	2016	S. Enteritidis	S	S	S	S	S	S	S	S	S	S	I	S	S	R	R	S
25	7378 conf. 7/3	2016	S. Enteritidis	S	S	S	I	S	S	S	S	n.t.	S	S	S	I	R	R	I
26	111640 CONF.6/1	2013	S. Typhimurium	I	S	n.t.	S	S	S	n.t.	n.t.	n.t.	S	n.t.	S	S	n.t.	S	S
27	111640 CONF.6/2	2013	S. Typhimurium	I	S	S	S	S	S	n.t.	n.t.	n.t.	S	n.t.	S	S	n.t.	S	S
28	111640 CONF.6/3	2013	S. Typhimurium	I	S	S	S	S	S	n.t.	n.t.	n.t.	S	n.t.	S	S	n.t.	S	S
29	93696	2013	S. Typhimurium	R	R	S	S	S	S	n.t.	S	n.t.	S	S	S	I	n.t.	S	I
30	114555 CONF. 4/3	2014	S. Typhimurium	S	n.t.	S	S	S	S	S	S	S	I	I	S	S	R	S	I
31	94778	2015	S. Typhimurium	S	I	S	S	S	S	S	S	S	S	S	S	S	R	I	S

n.	Lab cod.	YEAR	SUBTYPE or SEROTYPE	A	AMC	C	KF	CTX	CIP	CST	CAZ	ENR	G	K	NAL	S	SSS	SXT	T
33	94516	2015	S. Typhimurium	I	S	S	S	S	S	S	S	S	S	I	S	I	R	R	I
34	99713	2015	S. Typhimurium	I	S	n.t.	S	S	S	S	S	S	S	S	S	S	R	S	S
35	95927	2014	S. Typhimurium	S	S	S	I	S	S	S	S	n.t.	S	n.t.	S	S	R	R	I
36	95437	2014	S. Typhimurium monophasic variant 1,4,[5],12:I:-	R	R	S	R	S	S	S	S	n.t.	S	n.t.	S	R	R	R	R
37	95438 CONF.4/1-2	2014	S. Typhimurium monophasic variant 1,4,[5],12:I:-	R	R	S	R	S	S	S	S	n.t.	S	n.t.	S	R	R	R	R
38	95752	2014	S. Typhimurium monophasic variant 1,4,[5],12:I:-	R	R	S	R	S	S	S	S	n.t.	S	n.t.	S	R	n.t.	R	R
39	95445	2014	S. Typhimurium monophasic variant 1,4,[5],12:I:-	R	R	S	I	S	S	S	S	n.t.	S	n.t.	S	R	R	R	R
40	89846 CONF.4/1-2	2014	S. Infantis	S	S	S	I	S	S	S	I	n.t.	S	n.t.	S	S	R	S	I
41	89847 CONF.3/1-2	2014	S. Infantis	S	S	S	S	S	S	S	I	n.t.	S	n.t.	S	S	R	I	R
42	89848 CONF.3/1-2	2014	S. Infantis	S	S	S	R	S	S	S	I	n.t.	S	n.t.	S	S	R	I	I
43	91737 CONF. 4/1	2013	S. Newport	S	S	S	S	S	S	S	S	S	S	I	S	R	R	S	I
44	91737 CONF. 4/2	2013	S. Newport	S	S	S	S	S	S	S	S	S	S	I	S	R	R	S	I
45	91737 CONF. 4/3	2013	S. Newport	S	S	S	S	S	S	S	S	S	S	I	S	R	R	S	I
46	91737 CONF. 4/4	2013	S. Newport	S	S	S	S	S	S	S	S	S	S	I	S	R	R	S	I
47	91737 CONF. 4/6	2013	S. Newport	S	S	S	S	S	S	S	S	S	S	I	S	R	R	S	I
48	100063	2013	S. Newport	S	S	S	S	S	S	S	S	S	S	S	S	S	R	S	S
49	5547	2014	S. Newport	S	S	S	S	S	S	S	S	S	S	S	S	S	R	S	S
50	1613	2016	S. Newport	S	S	S	S	S	S	S	S	S	S	I	S	S	R	R	I
51	98945	2013	S. Napoli	S	S	S	S	S	S	S	S	S	S	S	S	S	R	S	S
52	99103	2013	S. Napoli	S	S	S	S	S	S	S	S	S	S	S	S	S	R	S	S
53	103159 CONF.6/1	2013	S. Napoli	R	S	n.t.	I	S	S	S	I	S	S	I	n.t.	I	R	S	S
54	103159 CONF.6/2	2013	S. Napoli	R	S	n.t.	I	S	S	S	I	S	S	I	n.t.	I	R	S	S
55	103197	2013	S. Napoli	S	S	S	S	S	S	S	S	S	S	S	S	S	I	S	S
56	92864	2013	S. Napoli	S	S	S	S	S	S	S	S	S	S	I	S	S	R	S	I
57	100413	2013	S. Napoli	R	S	S	S	S	S	S	I	S	I	I	S	I	R	S	S
58	91489	2013	S. Napoli	S	S	S	S	S	S	n.t.	I	S	S	S	S	S	R	S	R
59	93470	2013	S. Napoli	S	S	S	S	n.t.	S	n.t.	S	S	S	S	S	S	R	S	I
60	100437	2013	S. Napoli	S	S	S	S	S	S	S	S	S	S	S	S	S	I	S	S
61	109450	2013	S. Napoli	S	S	S	S	S	S	S	S	S	S	S	S	S	R	S	S
62	109452	2013	S. Napoli	I	S	S	S	S	S	S	S	S	S	S	S	S	S	S	S
63	111850	2013	S. Napoli	R	I	S	S	I	S	S	S	S	S	S	S	R	R	S	R
64	108435	2013	S. Napoli	I	S	S	S	S	S	S	I	S	S	I	S	S	R	S	S

n.	Lab cod.	YEAR	SUBTYPE or SEROTYPE	A	AMC	C	KF	CTX	CIP	CST	CAZ	ENR	G	K	NAL	S	SSS	SXT	T
66	103919	2013	S. Napoli	S	S	S	S	S	S	S	S	S	S	S	S	S	R	S	I
67	114555 CONF.2/4	2014	S. Napoli	S	S	S	S	S	S	S	S	S	S	S	S	S	R	S	S
68	114555 CONF. 3/2	2014	S. Napoli	S	I	S	S	S	S	S	S	S	I	I	S	S	R	S	S
69	55058	2014	S. Napoli	S	S	S	I	I	S	S	S	S	S	I	S	S	R	S	S
70	109263	2014	S. Napoli	S	S	S	S	S	S	S	S	n.t.	S	S	S	S	R	S	S
71	89697	2014	S. Napoli	S	S	S	S	S	S	S	S	n.t.	S	S	S	S	R	S	S
72	117148	2013	S.Coeln	S	S	S	S	S	S	S	S	S	S	S	S	S	R	S	S
73	93647	2013	S.Coeln	S	I	S	I	I	S	S	I	S	S	I	I	I	R	S	R
74	93655	2013	S.Coeln	I	R	S	R	I	S	R	R	I	I	I	n.t.	R	R	I	R
75	93695	2013	S.Coeln	S	I	S	I	S	S	I	I	I	S	I	S	I	R	S	I
76	59665 CONF. 8/1-2-3-4	2013	S.Coeln	R	I	S	I	I	S	S	S	I	S	n.t.	I	I	R	I	R
77	96997	2015	S.Coeln	I	R	S	S	S	S	S	S	S	S	S	S	S	R	I	S
78	97013	2015	S.Coeln	S	I	S	I	S	S	S	S	S	S	I	S	R	R	R	R
79	1633 CONF.4/2	2016	S.Coeln	I	S	S	S	S	S	S	S	S	S	I	S	S	R	R	R
80	7378 conf.4/5	2016	S.Coeln	S	S	S	S	S	S	S	S	S	S	I	I	S	R	I	R
81	97228 CONF. 7/1	2013	S. Brandebourg	R	R	I	R	R	S	I	I	I	n.t.	R	I	I	R	I	R
82	97228 CONF. 7/2	2013	S. Brandebourg	R	R	I	R	R	S	I	I	I	n.t.	R	I	I	R	I	R
83	97228 CONF. 7/3	2013	S. Brandebourg	R	R	I	R	R	S	I	I	I	n.t.	R	I	I	R	I	R
84	90111 CONF.3/12	2013	S. Oxford	I	S	S	S	S	S	S	S	S	S	S	S	S	R	S	S
85	90111 CONF.3/13	2013	S. Oxford	I	S	S	S	S	S	S	S	S	S	S	S	S	R	S	S
86	90111 CONF.3/14	2013	S. Oxford	I	S	S	S	S	S	S	S	S	S	S	S	S	R	S	S
87	90111 CONF.3/15	2013	S. Oxford	I	S	S	S	S	S	S	S	S	S	S	S	S	R	S	S
88	90111 CONF.3/16	2013	S. Oxford	I	S	S	S	S	S	S	S	S	S	S	S	S	R	S	S
89	90111 CONF.3/18	2013	S. Oxford	I	S	S	S	S	S	S	S	S	S	S	S	S	R	S	S
90	114555 CONF.7/1	2014	S. Oxford	S	n.t.	S	S	I	S	S	S	S	I	I	S	I	R	R	I
91	87963 CONF.4/1	2013	S. Stourbridge	S	S	S	S	S	S	S	S	S	I	I	I	R	R	R	R
92	87963 CONF.4/2	2013	S. Stourbridge	S	S	S	S	S	S	S	S	S	I	I	I	R	R	R	R
93	93700	2013	S. Stourbridge	S	I	S	I	S	S	S	I	S	S	I	I	I	R	S	I
94	83342 CONF.6/1	2013	S. Muenster	R	R	S	R	S	S	S	S	S	S	S	S	S	R	S	S
95	83342 CONF.6/2	2013	S. Muenster	S	S	S	S	S	S	S	S	S	S	S	I	S	R	S	S
96	83342 CONF.6/3	2013	S. Muenster	S	S	S	S	S	S	S	S	S	S	S	R	S	R	S	S
97	83342 CONF.6/4	2013	S. Muenster	I	S	S	I	S	S	S	S	S	S	S	S	S	R	S	S

n.	Lab cod.	YEAR	SUBTYPE or SEROTYPE	A	AMC	C	KF	CTX	CIP	CST	CAZ	ENR	G	K	NAL	S	SSS	SXT	T
99	102745	2013	S. Muenster	S	S	S	S	S	S	S	I	S	S	S	S	S	I	S	I
100	90720	2013	S. Atkpame	R	R	S	R	I	S	n.t.	S	n.t.	S	S	S	S	R	R	R
101	93703	2013	S. Goldocast	S	I	S	I	I	S	S	I	I	S	I	S	I	R	S	I
102	93740	2013	S. Kottbus	I	I	S	R	I	S	I	I	S	S	S	I	I	R	S	I
103	108931	2013	S. Kottbus	I	S	S	S	S	S	S	I	S	S	I	S	S	R	S	S
104	108933	2013	S. Kottbus	S	S	S	S	S	S	S	S	S	S	S	S	S	R	S	S
105	99687 CONF.4/1-2	2015	S. Kottbus	I	S	S	S	S	S	S	S	S	S	I	S	S	R	R	S
106	7378 conf. 10/1	2016	S. Kottbus	S	S	S	I	S	S	S	S	I	I	S	S	I	R	I	I
107	100469	2013	S. Thompson	S	S	S	S	S	S	S	S	S	S	S	S	S	S	S	S
108	103912	2013	S. Thompson	S	S	S	S	S	S	S	S	S	S	S	S	S	R	S	R
109	100908	2014	S. Thompson	S	S	S	S	S	S	S	S	S	S	S	S	I	n.t.	S	S
110	90437 CONF.4/2-4	2014	S. Thompson	S	I	S	I	S	S	S	I	n.t.	S	n.t.	S	R	R	S	R
111	90437 CONF.4/ CAMP.3	2014	S. Thompson	S	S	S	S	S	S	S	I	n.t.	S	n.t.	S	I	R	S	R
112	5791	2014	S. Thompson	S	S	S	S	S	S	S	S	S	S	S	S	S	R	S	S
113	5791	2014	S. Thompson	S	S	S	S	S	S	S	S	S	S	S	S	S	R	S	S
114	107000	2015	S. Thompson	S	S	S	S	S	S	S	S	S	S	I	S	S	R	I	S
115	90059 CONF.8/CAMP.2	2013	S. Veneziana	S	S	n.t.	I	S	S	n.t.	I	S	S	I	S	I	R	R	R
116	89108	2013	S. Veneziana	S	S	S	S	S	S	n.t.	S	S	S	S	S	S	R	S	S
117	103878	2013	S. Veneziana	S	S	S	S	S	S	S	S	S	S	S	S	S	R	S	S
118	103918	2013	S. Veneziana	S	S	S	S	S	S	S	S	S	S	S	S	S	R	S	I
119	2645	2014	S. Veneziana	S	S	S	S	S	S	S	S	S	S	S	S	S	R	S	S
120	107013	2015	S. Veneziana	S	S	S	S	S	S	S	S	S	S	I	S	S	R	S	S
121	7378 conf. 3/1	2016	S. Veneziana	S	I	S	S	S	S	S	S	S	I	S	S	S	R	R	R
122	7378 conf. 3/2	2016	S. Veneziana	S	S	S	S	S	S	S	S	S	S	I	S	S	R	I	R
123	7378 conf. 8/3	2016	S. Veneziana	S	I	S	S	S	S	I	I	I	R	R	I	R	R	R	I
124	7378 conf. 8/4	2016	S. Veneziana	S	S	S	S	S	S	S	S	I	R	R	I	I	R	R	R
125	114555 CONF. 4/ CAMP.10	2014	S. Stoneferry	S	n.t.	S	S	S	S	S	S	S	S	S	S	S	R	S	S
126	114555 CONF.5/ CAMP. 1	2014	S. Juba	S	n.t.	S	S	S	S	S	S	S	S	S	S	S	R	S	I
127	114555 CONF.5/ CAMP. 3	2014	S. Juba	S	n.t.	S	S	S	S	S	S	S	S	S	S	S	R	R	S
128	114555 CONF.5/ CAMP. 2	2014	S. Arechavaleta	S	n.t.	S	S	S	S	S	S	S	S	S	S	S	R	R	S
129	114555 CONF.5/ CAMP. 4	2014	S. Arechavaleta	S	n.t.	S	S	S	S	S	S	S	S	S	S	S	R	R	S
130	114555 CONF.8/ CAMP. 1	2014	S. Gail	S	S	S	S	S	S	S	S	S	S	S	S	S	R	I	S

n.	Lab cod.	YEAR	SUBTYPE or SEROTYPE	A	AMC	C	KF	CTX	CIP	CST	CAZ	ENR	G	K	NAL	S	SSS	SXT	T
132	114555 CONF.8/ CAMP. 4	2014	S. Gail	S	S	S	S	S	S	S	S	S	S	I	S	S	R	I	S
133	114555 CONF.8/ CAMP. 3	2014	S. Gail	S	S	S	S	S	S	S	S	S	I	I	S	S	R	I	S
134	114555 CONF.7/ CAMP. 2	2014	S. Kiumenza	S	n.t.	S	S	I	S	S	S	S	I	I	S	I	R	I	I
135	114555 CONF.7/ CAMP. 3	2014	S. Kiumenza	S	n.t.	S	S	I	S	S	S	S	I	I	S	I	R	I	I
136	114555 CONF.9/ CAMP. 1	2014	S. Kiumenza	S	S	S	S	I	S	S	I	S	R	I	S	I	R	S	I
137	114555 CONF.9/ CAMP. 2	2014	S. Kiumenza	S	S	S	S	S	S	S	S	S	S	I	S	S	R	I	S
138	114555 CONF.9/ CAMP. 3	2014	S. Kiumenza	S	S	S	S	S	S	S	I	S	I	I	S	I	R	S	S
139	114555 CONF.9/ CAMP. 4	2014	S. Kiumenza	S	S	S	S	S	S	S	I	S	I	I	S	S	R	S	S
140	114555 CONF.10/ CAMP.1	2014	S. Banjul	S	S	S	S	S	S	S	S	S	S	S	S	I	R	R	S
141	114555 CONF.10/ CAMP.2	2014	S. Banjul	S	S	S	S	S	S	S	S	S	S	S	S	S	R	R	S
142	114555 CONF.10/ CAMP.3	2014	S. Banjul	S	S	S	S	S	S	S	S	S	S	S	S	S	R	R	S
143	114555 CONF.10/ CAMP.4	2014	S. Banjul	S	S	S	S	S	S	S	S	S	S	S	S	S	R	R	S
144	92980	2014	S. Grampiam	S	S	S	I	S	S	S	S	n.t.	S	n.t.	I	I	R	I	R
145	2626	2014	S. Ablogame	S	S	S	S	S	S	S	S	S	S	S	S	I	R	S	S
146	1242	2014	S. Massakory	S	S	S	S	S	S	S	S	S	S	S	n.t.	S	R	S	S
147	104841 CONF.2/ CAMP.1-2	2015	S. Biespebjerg	S	I	S	I	S	S	S	S	S	S	I	I	S	R	S	I
148	7378 conf. 2/3	2016	S. Canada	R	S	S	S	S	S	S	S	S	S	I	S	S	R	R	R
149	7378 conf. 3/4	2016	S. Canada	S	S	S	S	S	S	S	S	S	S	I	S	I	R	R	R
150	7378 conf. 3/5	2016	S. Canada	S	S	S	S	S	S	S	S	S	S	I	S	S	R	R	R
151	7378 conf. 3/6	2016	S. Canada	S	S	S	S	S	S	S	S	S	S	I	S	I	R	I	R
152	7378 conf.4/2	2016	S. Canada	S	S	S	I	I	S	S	S	S	S	I	S	S	R	S	R
153	7378 conf. 5/4	2016	S. Canada	S	S	S	I	S	S	S	S	S	I	I	S	I	R	R	I
154	7378 conf. 5/5	2016	S. Canada	S	S	S	S	S	S	S	S	S	I	I	S	S	R	R	R
155	7378 conf. 6/2	2016	S. Canada	S	S	S	I	S	S	S	S	n.t.	S	I	S	S	n.t.	R	S
156	7378 conf. 8/1	2016	S. Bahrenfeld	S	S	S	S	I	I	S	S	I	R	R	I	I	R	R	R
157	89320 CONF.5/CAMP. 2	2013	S. Umbilo	R	S	S	S	S	S	S	I	S	S	I	S	S	R	I	R
158	85502	2013	S. enterica subs. salamae	R	S	S	I	S	S	S	I	S	S	I	I	I	R	S	S
159	103877	2013	S. enterica subs. salamae	S	S	S	S	S	S	S	S	S	S	S	S	S	R	S	S
160	91498	2013	S. enterica subs. salamae	R	R	R	R	S	S	n.t.	I	S	S	S	n.t.	n.t.	R	R	R
161	89320 CONF.5/CAMP. 1	2013	S. enterica subs. salamae	R	S	S	I	S	S	S	I	S	S	S	S	S	R	S	S
162	114555 CONF.2/ CAMP. 2	2014	S. enterica subs. salamae	S	S	S	S	S	S	S	S	S	S	S	S	S	R	S	S
163	114555 CONF.2/ CAMP. 3	2014	S. enterica subs. salamae	S	I	S	S	S	S	S	S	S	S	S	S	S	R	S	S

n.	Lab cod.	YEAR	SUBTYPE or SEROTYPE	A	AMC	C	KF	CTX	CIP	CST	CAZ	ENR	G	K	NAL	S	SSS	SXT	T
165	114555 CONF.2/ CAMP. 7	2014	S. enterica subs. salamae	S	I	S	S	S	S	S	S	S	S	S	S	S	R	S	S
166	114555 CONF. 3/ CAMP.1	2014	S. enterica subs. salamae	S	n.t.	S	S	S	S	S	S	S	I	S	S	S	R	I	S
167	114555 CONF. 3/ CAMP.10	2014	S. enterica subs. salamae	S	n.t.	S	S	S	S	S	S	S	S	S	S	I	R	S	S
168	114555 CONF. 3/ CAMP.11	2014	S. enterica subs. salamae	S	n.t.	S	S	S	S	S	S	S	S	S	S	I	R	S	S
169	114555 CONF. 3/ CAMP.12	2014	S. enterica subs. salamae	S	I	S	S	S	S	S	S	S	S	S	S	S	R	S	I
170	114555 CONF. 3/ CAMP.13	2014	S. enterica subs. salamae	S	n.t.	S	S	S	S	S	S	S	S	S	S	S	R	S	S
171	114555 CONF. 3/ CAMP.15	2014	S. enterica subs. salamae	S	n.t.	S	S	S	S	S	S	S	S	S	S	S	R	S	S
172	114555 CONF. 3/ CAMP.16	2014	S. enterica subs. salamae	S	n.t.	S	S	S	S	S	S	S	S	S	S	S	R	S	S
173	114555 CONF. 4/ CAMP.2	2014	S. enterica subs. salamae	S	n.t.	S	S	S	S	S	S	S	S	I	S	S	R	S	S
174	114555 CONF. 4/ CAMP.8	2014	S. enterica subs. salamae	S	n.t.	S	I	I	S	S	S	S	I	I	S	I	R	S	S
175	114555 CONF. 4/ CAMP.9	2014	S. enterica subs. salamae	S	n.t.	S	S	S	S	S	S	S	I	I	S	S	R	S	I
176	114555 CONF. 4/ CAMP.11	2014	S. enterica subs. salamae	S	n.t.	S	S	S	S	S	I	S	S	S	S	I	R	I	S
177	114555 CONF. 4/ CAMP.12	2014	S. enterica subs. salamae	S	n.t.	S	S	S	S	S	S	S	I	I	I	S	R	S	S
178	114555 CONF.5/ CAMP. 7	2014	S. enterica subs. salamae	S	n.t.	S	S	S	S	S	S	S	S	I	S	S	R	I	S
179	114555 CONF.5/ CAMP. 8	2014	S. enterica subs. salamae	S	n.t.	S	S	S	S	S	S	S	S	S	S	S	R	I	S
180	114555 CONF.5/ CAMP. 9	2014	S. enterica subs. salamae	S	n.t.	S	S	S	S	S	S	S	S	S	S	S	R	I	S
181	114555 CONF.6/ CAMP. 1	2014	S. enterica subs. salamae	S	n.t.	S	S	S	S	S	S	S	S	I	S	I	R	I	I
182	114555 CONF.6/ CAMP. 2	2014	S. enterica subs. salamae	S	n.t.	S	I	S	S	S	S	S	S	I	S	S	R	I	S
183	114555 CONF.7/ CAMP. 4	2014	S. enterica subs. salamae	S	n.t.	S	S	S	S	S	S	S	I	I	S	S	R	I	I
184	85139	2014	S. enterica subs. salamae	S	S	S	I	S	S	S	n.t.	n.t.	S	n.t.	S	I	R	S	I
185	85143	2014	S. enterica subs. salamae	R	S	S	R	S	S	S	S	n.t.	S	n.t.	I	S	R	S	R
186	85152	2014	S. enterica subs. salamae	R	S	S	I	S	S	S	S	n.t.	S	n.t.	I	S	R	S	R
187	85170 CONF.3/ CAMP.1-2-3	2014	S. enterica subs. salamae	S	I	S	R	I	S	S	I	n.t.	S	n.t.	I	I	R	I	R
188	85188 CONF. 4/ CAMP.1-2	2014	S. enterica subs. salamae	S	S	S	I	S	S	S	I	n.t.	S	n.t.	I	S	R	S	S
189	85190 conf.3/ CAMP. 1-2	2014	S. enterica subs. salamae	S	S	S	I	S	S	S	I	n.t.	S	n.t.	I	S	R	S	R
190	85191	2014	S. enterica subs. salamae	R	I	S	I	S	S	S	S	n.t.	S	n.t.	I	S	R	S	I
191	89407 CONF.3/ CAMP. 1-2	2014	S. enterica subs. salamae	S	S	S	S	S	S	S	I	n.t.	S	n.t.	S	S	R	I	S
192	89837 CONF.4/ CAMP.1-2	2014	S. enterica subs. salamae	S	S	S	I	I	S	S	R	n.t.	S	n.t.	S	S	R	I	R
193	89843 CONF.4/ CAMP.1-2	2014	S. enterica subs. salamae	S	S	S	I	S	S	S	S	n.t.	S	n.t.	I	S	R	S	I
194	90440	2014	S. enterica subs. salamae	S	S	S	S	S	S	S	S	n.t.	S	n.t.	I	S	R	S	I
195	101560	2014	S. enterica subs. salamae	S	S	S	S	S	S	S	S	S	S	S	S	S	R	S	S
196	101566	2014	S. enterica subs. salamae	S	S	S	I	S	S	S	S	S	S	S	S	S	R	S	S

n.	Lab cod.	YEAR	SUBTYPE or SEROTYPE	A	AMC	C	KF	CTX	CIP	CST	CAZ	ENR	G	K	NAL	S	SSS	SXT	T
198	114165	2014	S. enterica subs. salamae	S	S	S	S	S	S	S	n.t.	n.t.	S	I	S	S	R	S	S
199	108976	2014	S. enterica subs. salamae	S	S	S	S	S	S	S	S	n.t.	S	S	S	S	S	S	S
200	108979	2014	S. enterica subs. salamae	S	S	S	S	S	S	S	S	n.t.	S	S	S	S	R	S	S
201	2041	2015	S. enterica subs. salamae	S	I	S	I	S	S	S	I	n.t.	S	S	S	S	R	I	S
202	2045	2015	S. enterica subs. salamae	S	S	S	S	S	S	S	S	n.t.	S	S	S	I	R	I	I
203	2146	2015	S. enterica subs. salamae	S	S	S	S	S	S	S	I	n.t.	S	I	S	I	R	I	I
204	2147	2015	S. enterica subs. salamae	S	I	S	S	S	S	S	I	n.t.	S	I	S	S	R	I	S
205	7378 conf. 2 /1	2016	S. enterica subs. salamae	S	S	S	S	S	S	S	S	S	S	I	S	S	R	R	R
206	7378 conf. 2/4	2016	S. enterica subs. salamae	S	S	S	S	S	S	S	S	S	S	I	S	S	R	R	R
207	7378 conf. 6/1	2016	S. enterica subs. salamae	R	I	S	R	S	S	S	S	S	S	I	S	R	R	R	R
208	7378 conf. 8/2	2016	S. enterica subs. salamae	S	S	S	I	S	S	S	S	I	R	R	S	R	R	I	I
209	7378 conf. 9/1	2016	S. enterica subs. salamae	S	S	S	I	S	I	I	I	R	R	R	I	I	R	R	I
210	7378 conf. 9/2	2016	S. enterica subs. salamae	S	S	S	S	S	S	S	S	I	R	R	I	I	R	R	R
211	114555 CONF.2/1	2014	S. enterica subs. arizonae	S	n.t.	S	S	S	S	S	S	S	S	S	S	S	R	R	S
212	114555 CONF.2/8	2014	S. enterica subs. arizonae	S	n.t.	S	S	S	S	S	S	S	S	I	S	I	R	S	S
213	114555 CONF. 4/5	2014	S. enterica subs. arizonae	S	n.t.	S	S	S	S	S	S	S	I	I	S	I	R	I	S
214	114555 CONF. 4/6	2014	S. enterica subs. arizonae	S	n.t.	S	S	S	S	S	S	S	S	I	S	I	R	I	S
215	110305 CONF.6/1	2014	S. enterica subs. arizonae	S	S	S	S	S	S	S	S	S	S	S	S	R	R	S	S
216	110305 CONF.6/2	2014	S. enterica subs. arizonae	S	S	S	S	S	S	S	n.t.	n.t.	S	S	S	R	R	S	S
217	110310	2014	S. enterica subs. arizonae	S	S	S	S	S	S	S	n.t.	n.t.	S	I	S	I	R	S	S
218	110311	2014	S. enterica subs. arizonae	S	S	S	S	S	S	S	n.t.	n.t.	S	I	S	I	R	S	S
219	110363	2014	S. enterica subs. arizonae	S	S	S	S	S	S	S	n.t.	n.t.	S	S	S	S	R	S	S
220	111718 CONF.4/1-2-3	2014	S. enterica subs. arizonae	S	S	S	S	S	S	S	n.t.	n.t.	S	I	S	I	R	S	S
221	7378 conf. 6/3	2016	S. enterica subs. arizonae	I	I	S	I	S	S	S	S	S	S	S	S	I	n.t.	R	R
222	7378 conf. 7/1	2016	S. enterica subs. arizonae	S	S	S	I	S	S	S	S	S	S	I	S	I	n.t.	R	I
223	87962 conf.4/1	2013	S. enterica subs. diarizonae	S	S	S	S	S	S	S	S	S	S	S	S	I	R	R	S
224	87962 CONF.4/2	2013	S. enterica subs. diarizonae	R	S	S	I	S	S	S	I	I	S	I	I	I	R	S	I
225	91737 CONF.4/5	2013	S. enterica subs. diarizonae	I	S	S	I	I	S	S	I	S	S	R	n.t.	R	R	S	I
226	91737 CONF.4/7	2013	S. enterica subs. diarizonae	I	S	S	I	I	S	S	I	S	S	I	R	R	R	S	I
227	90718	2013	S. enterica subs. diarizonae	S	S	S	S	S	S	n.t.	S	S	S	I	S	R	R	R	I
228	103705	2013	S. enterica subs. diarizonae	S	S	n.t.	S	S	S	S	S	S	S	S	S	I	R	S	S
229	87963 CONF.5/1	2013	S. enterica subs. diarizonae	S	S	S	S	S	S	S	S	S	I	I	S	R	R	S	S

n.	Lab cod.	YEAR	SUBTYPE or SEROTYPE	A	AMC	C	KF	CTX	CIP	CST	CAZ	ENR	G	K	NAL	S	SSS	SXT	T
231	103373	2013	S. enterica subs. diarizonae	S	S	S	S	I	S	S	I	S	S	I	S	R	R	I	I
232	91506	2013	S. enterica subs. diarizonae	S	I	S	R	n.t.	S	S	I	S	S	I	S	n.t.	R	S	S
233	113172	2013	S. enterica subs. diarizonae	S	S	S	S	S	S	n.t.	S	n.t.	S	S	S	I	n.t.	S	S
234	114555 CONF. 3/14	2014	S. enterica subs. diarizonae	S	n.t.	S	S	S	S	S	S	S	S	I	I	S	R	S	S
235	114555 CONF. 4/7	2014	S. enterica subs. diarizonae	S	n.t.	S	S	S	S	S	S	S	S	S	S	I	R	S	S
236	114555 CONF.6/3	2014	S. enterica subs. diarizonae	S	n.t.	S	S	S	S	S	S	S	S	S	S	I	R	R	S
237	92885 CONF.4/2	2014	S. enterica subs. diarizonae	S	I	S	S	S	S	S	I	n.t.	S	n.t.	n.t.	R	R	R	R
238	92885 CONF.4/3	2014	S. enterica subs. diarizonae	S	S	S	S	S	S	S	S	n.t.	S	n.t.	S	I	R	S	S
239	2682	2014	S. enterica subs. diarizonae	S	S	S	S	S	S	S	S	S	S	S	S	I	R	S	S
240	2630	2014	S. enterica subs. diarizonae	S	I	S	S	S	S	S	S	S	S	S	S	I	R	S	S
241	2682	2014	S. enterica subs. diarizonae	S	S	S	S	S	S	S	S	S	S	S	S	I	R	S	S
242	110293	2014	S. enterica subs. diarizonae	S	S	S	S	S	S	S	n.t.	n.t.	S	S	S	I	R	S	S
243	103803	2014	S. enterica subs. diarizonae	S	S	S	S	S	S	S	S	n.t.	S	S	S	I	R	S	S
244	105516	2014	S. enterica subs. diarizonae	S	S	S	S	S	S	S	S	n.t.	S	S	S	S	R	S	S
245	90257	2015	S. enterica subs. diarizonae	I	S	S	S	S	S	S	I	S	S	I	S	I	R	I	R
246	93057 CONF.1/1-2-3	2015	S. enterica subs. diarizonae	I	S	S	S	S	S	S	I	n.t.	S	I	S	I	R	I	S
247	1633 CONF.4/1	2016	S. enterica subs. diarizonae	S	I	n.t.	I	S	S	S	S	S	S	I	S	S	R	R	R
248	79113	2016	S. enterica subs. diarizonae	I	S	S	S	S	S	S	S	I	I	I	S	R	R	S	R
249	7378 conf. 5/1	2016	S. enterica subs. diarizonae	S	S	S	S	S	S	S	S	S	S	I	S	S	R	R	I
250	7378 conf. 5/2	2016	S. enterica subs. diarizonae	S	S	S	S	S	S	S	S	S	I	R	S	I	S	R	I
251	7378 conf. 5/3	2016	S. enterica subs. diarizonae	S	I	S	I	S	S	S	S	S	S	I	S	S	n.t.	R	I
252	7378 conf. 7/2	2016	S. enterica subs. diarizonae	S	S	S	I	S	S	S	S	S	S	I	S	R	n.t.	R	R
253	90049	2013	S. enterica subs. houtenae	S	S	S	S	S	S	n.t.	S	S	S	I	S	I	R	R	I
254	111438	2013	S. enterica subs. houtenae	I	S	S	S	S	S	S	S	S	S	I	S	I	R	S	S
255	111424 CONF.6/1	2013	S. enterica subs. houtenae	S	S	n.t.	S	S	S	S	I	S	S	R	S	I	R	S	S
256	111424 CONF.6/2	2013	S. enterica subs. houtenae	S	S	n.t.	S	S	S	S	I	S	S	R	S	I	R	S	S
257	92902	2013	S. enterica subs. houtenae	S	S	S	S	S	S	n.t.	S	S	S	I	S	I	R	S	S
258	93493	2013	S. enterica subs. houtenae	S	S	S	S	S	S	n.t.	S	S	S	S	S	I	R	S	S
259	114555 CONF. 4/1	2014	S. enterica subs. houtenae	S	n.t.	S	S	S	S	S	S	S	S	S	S	I	R	S	S
260	114555 CONF.11/1	2014	S. enterica subs. indica	S	S	S	S	S	S	S	S	S	S	S	S	S	R	I	S

*n.t: sample not tested against the molecule
